# Integrating information and communication technology into nursing practice for resilience: A cross-sectional quantitative study

**DOI:** 10.1371/journal.pone.0324865

**Published:** 2025-06-04

**Authors:** Li-Fang Chang, Pi-Hsia Lee, Dawn Dowding, Chia-Jung Hsieh, Kuei-Ru Chou, Tso-Ying Lee

**Affiliations:** 1 Department of Medical Education, Taipei Medical University Hospital, Taipei, Taiwan; 2 School of Nursing, Taipei Medical University, Taipei, Taiwan; 3 Division of Nursing, Midwifery and Social Work, School of Health Sciences, University of Manchester, United Kingdom; 4 School of Nursing, National Taipei University of Nursing and Health Science, Taipei, Taiwan; 5 Nursing Research Center, Department of Nursing, Taipei Medical University Hospital, Taipei, Taiwan; Pontificia Universidad Catolica de Chile, CHILE

## Abstract

**Background:**

The adoption of information and communication technology (ICT) tools was accelerated by the COVID-19 pandemic to facilitate contactless interaction and reduce infection risk. However, there remains a lack of quantitative analysis regarding nurses’ perceptions and experience with these ICT tools.

**Purpose:**

This survey study aimed to assess nurses’ experience and satisfaction with ICT tools during the COVID-19 pandemic.

**Methods:**

Registered nurses were recruited into this questionnaire-based cross-sectional study. Questions about organizational ICT training and support, impact on patient care, and ICT perception were asked.

**Results:**

A total of 237 nurses completed and returned the questionnaire. The majority of nurses reported that ICT tools support instant consultation and communication with patients (62.0%). ICT tools’ suitability and effectiveness of features were 6.40 ± 1.72 and 6.10 ± 1.73, respectively. Nurses’ attitudes and perceived benefits of the ICT tools scored 3.59 ± 0.81 and 3.52 ± 0.87, respectively. The mean system usability score was 52.75 ± 11.75. Subgroup analyses highlighted the influence of ICT training, support, concerns about patient safety, and user anxiousness on the scores.

**Conclusion:**

Participants identified equipment, infrastructure, and workflow changes as major issues in ICT use, which require organizational efforts for improvement.

Addressing data utility and security concerns through policy initiatives is also imperative. This study highlights the necessity of organizational initiatives to promote good use of ICT tools and to enhance nursing resilience, especially when facing a health emergency, more resources should be allocated to the IT department that aims to respond soon; strategies including integrating digital tools for emergencies into other established systems, expanding the size of IT team for maintenance and providing training course with multiple modules.

## Introduction

The implementation of information and communication technology (ICT) in nursing has long been proposed to be the solution for facilitating telehealth [1], reducing healthcare costs [2], improving homecare nursing [3], and enhancing quality of healthcare, particularly in low-resource settings [4]. The implementation of ICT can compensate for labor shortage in clinical nursing [5], while nurses often have difficult to adopt ICT into nursing practice because of the lack of sufficient digital education in nursing schools, suggesting the importance on-the-job training for successful implementation of ICT in nursing practice [6].

The global COVID-19 pandemic represented an unprecedented crisis [7]. The accessibility of personal protective equipment (PPE) and the adoption of digital technologies was crucial for minimizing direct contact between healthcare professionals and patients [8,9]. The rapid adoption of ICT brought a significant shift in contactless patient care delivery by nurse in response to the COVID-19 pandemic [10]. Resilience is defined as the capacity to constructively adjust to challenging circumstances, which is particularly important for nursing practice [11]. Nurses had to quickly amend their work methods and patient interactions to incorporate ICT tools, including teleconferencing tools accessible through smart devices and personal computers to support virtual consultations [10,12], while it often required time and resources to get accustomed to [8]. The application of ICT has been emphasized as a means to accelerate progress in ensuring universal healthcare coverage and protection from health emergencies [13,14]. The necessity and importance of ICT utilization in nursing was accelerated by the COVID-19 pandemic [8,15].

ICT improves contemporary nursing practice, and technological embeddedness increases nurses’ confidences and nursing resilience [16]. However, there is very little structured evaluation of nurses’ experiences and perceptions of ICT usage in the healthcare setting during the COVID-19 pandemic. The present study aimed to explore the nurses’ perceptions and experiences with ICT adoption in their work environment during the COVID-19 pandemic, along with 5 potential influencing factors including effectiveness, tool suitability, benefits of the tools, attitude toward ICT, and the system usability scale (SUS). The anticipated results may help enhance nursing resilience.

### Background

Despite numerous studies exploring the acceptance, effectiveness, and efficiency of digital care technologies, there are wide variations in study methods, target settings, and target groups [17]. Research from the past decade mainly focused on nurses’ leadership, soft skills, training, and management in relation to their digital knowledge, and new methodological approaches have also been pivotal in advancing the profession’s readiness to embrace telemedicine [18]. Prior to the COVID-19 crisis, healthcare professional knowledge and skills in ICT have been already identified as a key competence area for healthcare digitalization [19]. During the COVID-19 pandemic, a retrospective, qualitative analysis of nurses’ activity diaries identified communication difficulties with either colleagues or patients inside and outside the isolation units as key areas where ICT could offer support [20]. Information extracted from interviews between nurses and informatics specialists reveals that ICT facilitates non-contact communication and provision of emotional support for patients [21]. Moreover, human factors, such as knowledge and workflow issues regarding ICT use planning and evaluation, are often mentioned in interviews of nurses working in different sectors [22]. Hence, understanding the characteristics and preferences of the nursing workforce is pertinent to the successful implementation of ICT in nursing practice [23].

In addition, several barriers and facilitators of digital technology use have been found to be related to nurses’ work and operations [24]. A recent study revealed that nurses recognized access to support, resources, systems, and connectivity as barriers to ICT use during the pandemic, whereas ICT use promoted nurses’ access to colleagues and patients [25]. Dowding et al. (2023) found that although digital systems supported effective patient care, the usability of different systems varied significantly [26]. Furthermore, a systematic review indicated a notable gap in measuring the impact of digital health implementation during the pandemic, particularly in nursing and in a quantitative manner [27].

Factors associated with ICT adoption can be grouped into two categories as individual and organizational factors. Demographic factors such as age, gender, education level, in addition to personal factors, like competence, skills, experience, attitude and perception are both considered the individual factors that impact behavior of the ICT users and further ICT adoption. Organizational factors include the availability, security, reliability and ease of use of ICTs. For ICT adoption in healthcare, a multinational survey indicated that the individual factors were highly correlated to organizational performance, however, the direct impact of individual factors on ICT adoption was limited [28]. Similar findings have been found in other fields/scenarios. Analysis of a survey for ICT adoption within households indicated that gender, education, and place of residence did not affect the adoption of ICT significantly. The main factors were the economic status of households, cost, technological availability and security of ICTs, in addition to ICT competence and awareness, perceived economic benefits from the usage of ICT, and satisfaction with ICT adoption [29], which the organizational factors were involved. A study focusing on ICT adoption among Micro, Small, and Medium Enterprises (MSMEs) in India found that perceived usefulness and social influence were the dominating influence factors [30], which demonstrate the perception of ICT users and the interplay of these factors belonging to different categories may be valued to further improve the implementation of ICT.

Hence, this study aimed to investigate nurses’ perceptions of the ICT tools utilized in their work environment during the COVID-19 crisis using quantitative measures.

## Materials and methods

### Study design and participants

This study adopted a questionnaire-based, cross-sectional design and recruited nursing staff working in Taipei Medical University Hospital that is the first affiliated hospital of Taipei Medical University, Taipei, Taiwan by purposive sampling during Sep, 2023 to Nov. 2023. Registered nurses, who had been employed for at least 3 months and were able to use ICT tools independently in their work environments, were included in this study. Interns and probationary nurses were excluded. Despite the COVID period (2020−2022), our hospital still implemented the tight regulation of restrictive practice in 2023. So, participating nurses followed the same COVID-19 restrictions in 2023.

G*Power was used to calculate the required minimum total sample size in this study. The parameters for sample size estimation were set as: medium effect size: 0.3, confidence level: 95%, power: 0.8. The required minimum total sample size was 216. Assuming the attrition rate of 20%, a total of 270 questionnaires were sent out.

### Ethics

The study protocol was approved by the institutional review board of Taipei Medical University hospital (No. 202302002), and all participants provided informed consent prior to participating in the study.

### Instrument

We adopted the questionnaire developed by Dowding et al. (2023), which addresses the 7 domains of non-adoption, abandonment, scale-up, spread, and sustainability (NASSS) [26]. A Chinese version of the questionnaire, which has been scrutinized for cross-cultural adaptation including translation, synthesis, back translation, expert review (by 3 experts), pre-testing, and finalization, was adopted for the present study.

The original questionnaire contained questions regarding participant demographics (e.g., age, level of education, place of work, years of practice, etc.), ICT usage in their nursing work during the COVID-19 pandemic (each participant can rate up to 3 types of ICT of their choice), organizational ICT training and support, impact on patient care, and ICT perception (rated for feature effectiveness, tool suitability, beliefs that the benefits of the tools outweigh the risks, attitude toward ICT, and the SUS) [26]. The SUS score was derived from a set of 10 questions on the usability of the ICT, each rated using the 5-point Likert scale, and the maximum SUS score was 100. A score of 71 and above indicates good usability, while 51 and below indicates poor usability [31]. The benefit of ICTs used over the drawbacks was rated by the 5-point Likert scale (from strongly agree to strongly disagree). Similarly, the current attitude toward ICT was scaled using the 5-point Likert scale (from extremely positive to extremely negative). In addition, the effectiveness and suitability of ICTs used were scaled from 0 to 10.

In addition, the questionnaire contained 3 open-ended questions asking participants to identify potential factors influencing ICT use, problems related to ICT use that may arise in the future, and their specific concerns with increased ICT use. The questions have been reviewed and revised by 3 experts. Particularly, one of them has years of experience in developing rating scales using qualitative methods, and publishing qualitative articles [32]. The responses to these 3 questions could be classified to generate qualitative categories or items for rating scales as previously described [33]. The percentage of each category or item was calculated.

### Statistical analysis

All statistical analyses were performed using IBM SPSS software (version 22.0). Results of descriptive analyses are presented as the number of patients, percentage, mean, and standard deviation. The relationship between quantitative measures of ICT perception was examined with Pearson’s correlation. Post-hoc comparisons were conducted with the Scheffe’s or Dunnett’s T3 method. A p value of 0.05 and below indicated statistical significance.

## Results

### Participant demographics

Among 270 eligible participants, 237 registered nurses completed questionnaires (response rate 88%). Participants were aged between 24–64 years, with a mean age of 32.34 ± 8.60 years (Table 1). The majority of the participating nurses had a university degree or above (94.5%). The most common working place during the COVID-19 pandemic was outpatient clinic (42.6%), followed by general ward (23.2%), emergency room (13.5%), and COVID-19 ward (11.4%) (Table 1).

**Table 1:** Participant characteristics and their perception of ICT training, institutional support, and effect on patient care.

Item	Mean±SD or *n* (%)
**Characteristics**	
Total number of participants	237
Age (years)	32.34 ± 8.60
Nursing experience (years)	8.00 ± 7.59
Level of education	
College	13 (5.5%)
University or higher	224 (94.5%)
Unit of work	
Telemedicine service center	7 (3.0%)
Outpatient clinic	101 (42.6%)
General ward	55 (23.2%)
COVID-19 ward	27 (11.4%)
COVID-19 ICU	15 (6.3%)
Emergency room	32 (13.5%)
**Perception of ICT training, institutional support, and effect on patient care**	
Have you received sufficient training for the ICT being used?	
Yes	56 (23.6%)
Somewhat	92 (38.8%)
No	89 (37.6%)
Have your colleagues received sufficient training?	
Yes	73 (30.8%)
Somewhat	96 (40.5%)
No	68 (28.7%)
Is ICT support available when there is a problem?	
Most of the time	103 (43.5%)
Some of the time	91 (38.4%)
Rarely	43 (18.1%)
Has the use of ICT increased your workload?	
Yes	139 (58.6%)
No	98 (41.4%)
Have you provided feedback after using the ICT?	
Yes	46 (19.4%)
No	80 (33.8%)
Not sure	111 (46.8%)
Does the ICT fulfill the needs of patients?	
Most of the time	101 (42.6%)
Some of the time	119 (50.2%)
Rarely	17 (7.2%)
Are you concerned about the ICT-involved care provided to patients?	
A lot	8 (3.4%)
A moderate amount	43 (18.1%)
A little	106 (44.7%)
No	80 (33.8%)
Do you consider the ICT-involved care received by patients to be safe?	
Yes	106 (44.7%)
Somewhat safe	119 (50.2%)
No	12 (5.1%)
Will you continue to use the ICT in your work?	
Yes	118 (49.8%)
Maybe	106, (44.7%)
No	13 (5.5%)
Does the ICT comply with your workflow?	
Most of the time	84 (35.4%)
Some of the time	115 (48.5%)
Rarely	38 (16.0%)
Does the use of ICT make you feel anxious?	
Yes	91 (38.4%)
No	146 (61.6%)
How often do you encounter ICT-related problems needing workarounds?	
Always	30 (12.7%)
Most of the time	87 (36.7%)
Half of the time	18 (7.6%)
Some of the time	76 (32.1%)
Never	26 (11.0%)

ICT, information and communication technology; ICU, intensive care unit; SD, standard deviation.

### Characteristics of ICT being used

Some ICT tools that had already implemented prior to the COVID-19 crisis included telemedicine consultation (16.5%), data conversion tools for wearable devices (54.4%), and AI-assisted prediction tools (6.3%) (Table 2). Some ICT tools that were implemented during the pandemic, included teleconferencing or patient video-monitoring (31.2%), instant messaging or social apps (31.2%), electronic information and statement sheets for travel, occupation, contact, and cluster history (TOCC) (79.3%), and geofencing (24.9%). Particularly, telemedicine consultation tools and AI-assisted prediction tools were solely utilized in the telemedicine service center and the COVID-19 ward, respectively. In contrast, other ICT tools were used in multiple work environments. The features of the ICT tools are summarized in **Table 2**.

**Table 2:** Summary of ICT characteristics.

Item	*n*	% of total participants (N = 237)
ICT used		
Telemedicine consultation tool (already in place)^a^	39	16.5%
Wearable device data conversion tool (already in place)^b^	129	54.4%
AI-assisted prediction tool for sepsis (already in place)^c^	15	6.3%
Teleconferencing or patient video-monitoring (new to organization)^d^	74	31.2%
Instant messaging or social apps (new to organization)^e^	74	31.2%
Electronic TOCC information and statement sheet (new to organization)^f^	188	79.3%
Geofencing (new to organization)^g^	59	24.9%
Target users		
Nurses	215	90.7%
Doctors	180	75.9%
Administrative staff	98	41.4%
Patient	98	41.4%
Carer	77	35.5%
What feature(s) does the ICT have?		
Allows patients to enter their own information or data	50	21.1%
Enables sharing of clinical information between organizations	94	39.7%
Enables remote patient monitoring	134	56.5%
Supports instant consultation/communication with colleagues	89	37.6%
Supports instant consultation/communication with patients	147	62.0%
Provision of prescriptions and medication management	62	26.2%
Supports decision making	26	11.0%

Where the ICT tools were used: ^a^Telemedicine service center; ^b^general ward and COVID-19 ward; ^c^COVID-19 ward; ^d^COVID-19 intensive care unit (ICU); ^e^COVID-19 ward, COVID-19 ICU, and emergency room (ER); ^f^outpatient clinic, general ward, and ER; ^g^COVID-19 ICU and ER.

ICT, information and communication technology; TOCC, travel, occupation, contact, and cluster history.

### Participant response to ICT training, institutional support, and effect on patient care

Most participants responded that the training they received for ICT was somewhat sufficient (38.8%), while the training their colleagues received was somewhat sufficient (40.5%) (Table 1). Most participants found ICT support to be available “most of the time” (43.5%), but also that the use of ICT had actually increased their workload (58.6%). The participants felt that use of ICT fulfilled the needs of patients “some of the time” (50.2%) or “most of the time” (42.6%). Most participants believed that the ICT-involved care was “somewhat safe” (50.2%) or “safe” (44.7%) to the patients. Most participants were willing (49.8%) or might be willing (44.7%) to continue using ICT tools, as they found that ICT complied with their workflow “some of the time” (48.5%) or “most of the time” (35.4%). Nevertheless, 36.7% of participants expressed that problems needing workarounds were encountered “most of the time.” (Table 1)

### ICT perception and the influencing factors

With regards to the quantitative measures of ICT perception, ratings for ICT tools’ suitability for the work environment and effectiveness of features averaged 6.40 ± 1.72 and 6.10 ± 1.73, respectively (Table 3). The mean SUS was 52.75 ± 11.75. Nurses’ attitudes towards and perceived benefits of the ICT tools scored 3.59 ± 0.81 and 3.52 ± 0.87, respectively. All quantitative measures for ICT perception were positively correlated with each other (all *p* < 0.001), with a strong correlation observed between tool suitability and feature effectiveness (*r* = 0.76) and between beliefs of benefits and attitude (*r* = 0.71).

**Table 3:** The relationship between the different scales of ICT acceptance.

Variables	Mean±SD (min–max)	Correlation coefficient				
		Effectiveness	SUS	Suitability	Benefits	Attitude
Effectiveness	6.4 ± 1.72 (1–10)	1	0.45***	0.76***	0.41***	0.41***
SUS	52.75 ± 11.15 (1–100)	0.45***	1	0.47***	0.41***	0.44***
Suitability	6.10 ± 1.73 (1–10)	0.76***	0.47***	1	0.48***	0.46***
Benefits	3.59 ± 0.81 (1–5)	0.41***	0.41***	0.48***	1	0.71***
Attitude	3.52 ± 0.78 (1–5)	0.41***	0.44***	0.46***	0.71***	1

*** *p* < .001 by Pearson’s correlation. ICT, information and communication technology; max, maximum; min, minimum; SD, standard deviation; SUS, system usability scale.

Upon subgroup analysis, ICT training, support, concerns about patient safety, and anxiousness were observed to be associated with ICT perception (Table 4). Notably, participants who self-reported having at least some ICT training or receiving support at least some of the time gave higher scores for almost all of the perception measures compared to their counterparts. Those who considered the tools to be at least somewhat safe for patient care and reported not being anxious about using ICT were also more satisfied with the ICT tools compared to other participants.

**Table 4:** The association between ICT-related training, support, patient safety, and anxiousness with participant perception.

Subgroup (no. of patients)	Perception measure (mean ± standard deviation)				
	Effectiveness	SUS	Suitability	Benefits	Attitude
**Training**					
1. Yes (n = 56)	7.14 ± 1.63	60.09 ± 12.87	6.89 ± 1.52	3.95 ± 0.84	3.89 ± 0.73
2. Somewhat (n = 92)	6.48 ± 1.46	52.07 ± 8.79	6.20 ± 1.72	3.53 ± 0.81	3.47 ± 0.78
3. No (n = 89)	5.84 ± 1.85	48.85 ± 9.99	5.49 ± 1.65	3.44 ± 0.72	3.34 ± 0.75
F	10.78***	20.73***	12.63***	7.71**	9.61***
Post-hoc comparison	1 > 3, 2 > 3	1 > 2, 1 > 3^a^	1 > 2, 1 > 3, 2 > 3	1 > 2, 1 > 3	1 > 2, 1 > 3
**Support**					
1. Most of the time (n = 103)	7.00 ± 1.50	54.88 ± 11.70	6.74 ± 1.50	3.83 ± 0.79	3.79 ± 0.82
2. Some of the time (n = 91)	6.44 ± 1.70	52.03 ± 11.12	5.90 ± 1.80	3.46 ± 0.78	3.40 ± 0.71
3. Rarely (n = 43)	5.49 ± 1.78	49.19 ± 8.69	4.98 ± 1.44	3.33 ± 0.78	3.14 ± 0.60
F	14.83***	4.39*	19.29***	8.35***	13.43***
Post-hoc comparison	1 > 2, 1 > 3	1 > 3	1 > 2, 1 > 3, 2 > 3	1 > 2, 1 > 3	1 > 3, 2 > 3^a^
**Patient safety**					
1. Yes (n = 106)	7.39 ± 1.27	57.13 ± 11.57	6.86 ± 1.60	3.92 ± 0.78	3.79 ± 0.75
2. A little (n = 119)	5.77 ± 1.50	49.83 ± 8.86	5.64 ± 1.42	3.39 ± 0.71	3.34 ± 0.72
3. No (n = 12)	3.83 ± 1.85	43.13 ± 13.02	3.92 ± 2.23	2.75 ± 0.62	2.83 ± 0.84
F	56.81***	19.29***	29.92***	23.10***	15.72***
Post-hoc comparison	1 > 2, 1 > 3, 2 > 3	1 > 2, 1 > 3^a^	1 > 3, 1 > 3, 2 > 3	1 > 3, 1 > 3, 2 > 3	1 > 2, 1 > 3
**Anxiousness**					
Yes (n = 91)	6.02 ± 1.59	49.95 ± 12.03	5.53 ± 1.59	3.44 ± 0.83	3.46 ± 0.75
No (n = 146)	6.63 ± 1.77	54.50 ± 10.22	6.45 ± 1.72	3.69 ± 0.78	3.55 ± 0.81
T value	2.68	3.12	4.14	2.37	0.89
P value	0.008	0.002	<0.001	0.02	0.37

***p < 0.001, **p < 0.01, *p < 0.05. a, Dunnett T3 test.

ICT, information and communication technology; SUS, system usability scale.

The SUS score of ICT tools varied among different work environments, with participants from the telemedicine center being most satisfied about the usability of telemedicine consultation tools used in their work (72.14 ± 12.86 points) (Table 5). In contrast, participants from outpatient clinics were least satisfied with the electronic TOCC sheet used in their work (49.98 ± 9.29 points).

**Table 5:** SUS results stratified by work unit.

Variable	1. Telemedicine center^a^ (n = 7)	2. Outpatient clinic^b^ (n = 101)	3. General ward^c^ (n = 55)	4. COVID-19 ward^d^ (n = 27)	5. COVID-19 ICU^e^ (n = 15)	6. ER^f^ (n = 32)	F statistics
SUS (mean±SD)	72.14 ± 12.86	49.98 ± 9.29	53.77 ± 11.77	54.91 ± 11.67	55.33 ± 9.44	52.50 ± 10.98	6.67^***^

*** *p* < 0.001. Post-hoc comparison by Scheffe method (1 > 2, 1 > 3, 1 > 4, 1 > 5, 1 > 6).

ICT tools used: ^a^telemedicine consultation technology; ^b^electronic travel, occupation, contact, and cluster history (TOCC) sheet; ^c^wearable device data conversion tool and electronic TOCC sheet; ^d^wearable device data conversion tool, AI-assisted prediction tool, and instant messaging or social apps; ^e^teleconferencing or patient video-monitoring, instant messaging or social apps, and geofencing; ^f^instant messaging or social apps, electronic TOCC sheet, and geofencing.

ER, emergency room; ICT, information and communication technology; ICU, intensive care unit; SD, standard deviation.

### Open-ended questions

**Fig 1:**
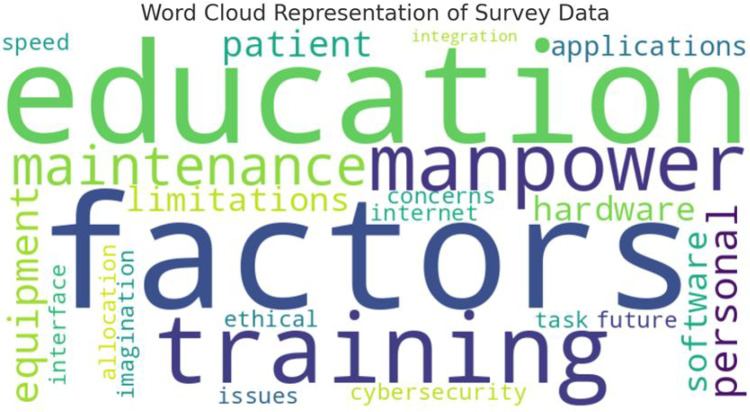
The word frequencies in open-ended questions.

Of the 85 participants who responded to the question regarding influencing factors, 10% (n = 9) identified factors related to staff, 53% (n = 45) identified factors related to equipment and hardware, and 27% (n = 23) identified factors related to patients. Among the 81 participants who provided answers to potential problems, 24% (n = 20)stated problems related to software and hardware availability, 22% (n = 18) stated data security, 14% (n = 11) stated the allocation and change in scope of work, 13% (n = 11) stated ethical issues, 8% (n = 7) stated training, 6% (n = 5) stated human resources and maintenance, and 14% (n = 11) stated the problems to be unsure. Of the 13 participants who addressed their specific concerns, 40% (n = 5) were worried about internet or network stability, 30% (n = 4) were worried about the integration between the various tools, and 30% (n = 4) were worried about maintenance. In addition, the percentages of responses to open-ended questions are presented in a visual word cloud (Fig 1).

## Discussion

The utilization of ICT in nursing practice advanced rapidly during the COVID-19 pandemic. This survey study revealed that the ICT tool most supported by nurses was instant consultation/communication with the patient, which enabled remote interactions and avoids face-to-face contact thereby reducing the risk of infection. This feature was available through instant messaging tools, social apps, and teleconferencing software used by the participants. Most participants considered the training they received somewhat sufficient. This perception may be attributed to time constraints and social-distancing rules implemented at the time. Given that sufficient training was observed to be associated with higher ICT satisfaction, we recommend implementing hybrid education modules to overcome time and space restrictions and enhance learning efficiency, both in the post-pandemic “new normal” and in future crisis situations. In line with our view, previous studies have highlighted the importance of including telenursing education in the nursing curriculum to develop digital competencies [34,35].

ICT has been used to enhance quality of healthcare in various settings decades ago. Due to the incredible advancement of information technology in the last two decades, Healthcare 4.0, a data-driven approach that integrates the Internet of Things, medical Cyber-Physical Systems, big data analysis, and machine learning, has been recently proposed to improve healthcare systems [36,37]. However, the extent of implementation of ICT varies between Europe and Africa, mainly depending on resources [4,5]. Taipei Medical University Hospital has invested a lot effort in building information technology infrastructure, and its big data center possesses the certification of “Smart Hospital” in Taiwan. Therefore, Taipei Medical University Hospital is suitable for conducting the present study in Taiwan.

The findings of this pilot study highlight the necessity of organizational initiatives to promote good use of ICT tools, thereby strengthening nursing resilience to challenges. Although the COVID-19 pandemic is over now, hospital administrators and policymakers should allocate more resources to constantly improve ICT infrastructure for better healthcare systems because ICT is rapidly evolving. However, real-world data collected from a hospital in Taiwan are required to persuade hospital administrators and policymakers to invest on-the-job training and ICT-assisted nursing care. Thus, this Taiwanese study provided insights into the successful implementation of ICT in healthcare system in Taiwan.

In order to evaluate health technology, the NASSS framework considers 7 domains: (i) condition, (ii) technology, (iii) value proposition, (iv) adopter system, (v) organization, (vi) wider system, and (vii) embedding and adaptation over time [38,39]. In the present study, participants viewed that the *condition* and *technology* aspects of the NASSS framework were partially met. ICT-related problems needing workarounds were encountered by almost 90% of respondents. Thus, some *technology*-related issues remain to be closely inspected. Regarding the *adopter system*, influencing factors related to staff and patients were mentioned in the responses to open questions. Training and support received by the respondents influenced their satisfaction with ICT tools, indicating that *organizational* efforts may be lacking in these aspects. In the wider system, ethical issues related to the utility and security of data require attention. The observation that telemedicine service centers staff were more satisfied with ICT tools in their work underscores the importance of *continuous embedding and adaptation over time* for successsful ICT implementation. In addition, the respondents were concerned about the change in the scope of work, emphasizing the need for long-term planning and adaptation. Similarly, the NASSS framework has been recently applied to identify challenges for ICT adoption in German outpatient healthcare [40].

Our findings also revealed that use of ICT could increase the nurse workload. One possible reason could be the process of taking and reporting TOCC information. The electronic TOCC sheet and reporting system was hastily developed and frequently updated to adapt to the rapidly changing COVID-19 landscape, including the constantly revising regulations at the time. While a notable proportion of study participants reported received ICT support most of the time, a higher number expressed at least some concerns about the care delivered to patients using these tools or were unsure about the safety of patients receiving such care. This suggests that the nursing staff did not view the total replacement of face-to-face clinical examinations, monitoring, and care with remote monitoring or telehealth as achievable [10].

In several aspects, the results of the present study highlight that organization-driven involvement may play a major role in ICT adoption. Participants who perceived had sufficient training and support had a higher level of satisfaction, a better perception of ICT, and less anxiety; in addition, SUS has a strong positive correlation between feature effectiveness and belief of benefits, respectively. The results indicate the necessary of more investment of ICT infrastructure building, training, and planning. The preparedness and availability of funds for ICT infrastructure building is reported as the most crucial factor for ICT adoption in previous study [41]. Despite the perceived usefulness being the pivotal factor for ICT adoption, significant barriers about resource/infrastructure, human resource, and technological environment that hinder ICT adoption should be overcome first [30]. Furthermore, individual attributes were found less inference on ICT adoption in healthcare, thus, increased user participation and collaboration with the IT department may be important [28], which are both organization-initiative strategies. In addition, individual factors such as attitude, competence, skill and knowledge about using digital tools can be improved easily only in an organization with a culture that is openness to change, innovation, and technological development since the implementation of sufficient training programs and open communication achieve positive perceptions of ICT [42]. Based on these findings, organization involvement should be engaged on ICT adoption proactively, especially when facing a health emergency like COVID-19 pandemic since quick response and resilience is urgent needed. Particularly, more resource should be allocated into the IT department as soon as possible to integrate the digital tools for emergency into other established systems such as electronic medical records system, to expand the size of the IT team for digital system maintenance, and to provide training course with multiple modules to fit the needs of nurses who may have time and space restrictions to receive sufficient training for ICT.

This study also revealed that participants held a moderately positive view of ICT being used in their work during the pandemic. Although ICT tools were employed to minimize direct contact, the physical presence of nursing staff may still be necessary in certain circumstances – such as making adjustment to devices and resolving signal issues. This requires nurses to don and doff PPE, likely increasing their emotional stress and workload [15]. The observation that a better perception of ICT was associated with sufficient training, adequate support, safer patient care, and not feeling anxious underscores the importance of education and the four components of the Schlossberg’s Transition Theory (situation, self, support, and strategy) [43]. Hence, we recommend considering a comprehensive plan to support, provision, and pilot personnel training to facilitate effective implementation of new ICT tools. This approach will enhance user acceptance and adaptability, ultimately improving the quality of care and reducing the burden on nursing staff during crisis situations.

In this study, participants from the telemedicine service center gave the highest SUS scores, while those from the outpatient clinic gave the lowest SUS scores. This finding could be partly explained by the types of ICT used and the patient volumes in different settings. Outpatient clinics typically handle a high volume of patients, along with their accompanying family members, who may require assistance with filling out the newly developed electronic TOCC sheets. In contrast, nursing staff at the telemedicine service center are likely already familiar with the telemedicine consultation tools used there, and interruptions or patient pressures may be minimized due to the nature of the service provided. Therefore, we highly recommend the continued expansion of telemedicine services, aligning with sustainability measures advocated by the WHO [13]. Coincidentally, the Ministry of Health and Welfare, Taiwan has proposed a budget of NTD 200,000,000 for the development of telehealth services and remote care following the easing of the COVID-19 regulations [44].

### Limitations and future research

There are several important limitations to acknowledge. First, due to the study design and subject recruitment approach, registered nurses were exclusively recruited from a single hospital. Further large-scale multicenter studies are warranted to confirm the current single-institute findings on resilience for health emergencies, thereby improving the generalizability. Second, the rated ICT encompasses multiple features, and participants did not specify which tool or feature they were referring to in their ratings. As a result, the current findings offer a general and amalgamated perspective on how nurses perceive the implementation of ICT in their work during the pandemic.

Notably, the research of artificial intelligence (AI) and its real-world applications has advanced remarkably in the past decade. AI has been proposed to be integrated into most aspects of life, including nursing care [45,46]. In the future, a longitudinal study will be conducted to explore the effects of the integration of AI and ICT on nurses’ experiences and perceptions of ICT usage, as well as quality and costs of nursing care.

## Conclusions

Participating nurses had a moderately positive perception and attitude toward ICT use during the COVID-19 pandemic, and generally considered the ICT tools were suitable and effective. However, nurses also encountered challenges and expressed concerns regarding training, support, the work platform, and infrastructure associated with ICT usage. These findings underscore the need for organizational efforts to address these issues and provide adequate support to nurses in utilizing digital technologies effectively, which will promote nurses’ confidences and help build resilience for health emergencies.

## Supporting information

S1 Data Supplementary data. (XLSX)
